# Cutaneous manifestations of Sin Nombre virus in the upper Midwest region

**DOI:** 10.1016/j.jdcr.2026.09.036

**Published:** 2026-09-26

**Authors:** Claire Diede, Brianne Haskell-Hanisch, Dallin Griffin, Jonathan E. Cutlan, Erik J. Stratman

**Affiliations:** a University of North Dakota School of Medicine, Grand Forks, North Dakota; b Department of Dermatology, Marshfield Clinic Health System, Marshfield, Wisconsin

**Keywords:** cutaneous manifestations, Hantavirus, purpura, rash, Sin Nombre virus, vector-borne illness

## Introduction

Sin Nombre virus (SNV) is the primary New World Hantavirus that causes Hantavirus pulmonary syndrome (HPS).^1^^,^^2^ The SNV virus was first documented in the Southwest region of the United States in 1993 and is carried by the vector *Peromyscus maniculatus*, or deer mouse.^1^ Hantaviruses are classified into 2 groups: New World, located in North and South America, and Old World, located in Europe, Africa, and Asia.^2^^,^^3^ Hantavirus infections are exceedingly rare in the upper Midwest, and typically involve patients returning from travel to more endemic areas. Herein we report a case of SNV infection in a patient with exposure to mice and no recent travel outside of Wisconsin, suggesting a potentially serious emerging infectious disease to the region.

## Case presentation

A 38-year-old previously healthy Hispanic male carpenter presented with progressive fever, malaise, chills, nausea, headache, diarrhea, bilateral conjunctival injection, and rash. Systemic symptoms began 5 days prior with symptom progression prompting medical attention. His asymptomatic rash began on his feet on day 3 of the febrile illness and had progressed in a caudocranial manner. History was negative for tick bites, beaver exposures, medications, or known exposure to ill people. Patient reported his only travel from his home in central Wisconsin was a trip the week prior with his family to northern Wisconsin where he reported petting zoo animal exposures, stream water splash exposure, and multiple mosquito bites. He reported encountering many recent live mice in his work environment in the weeks prior during various home remodeling jobs. On physical examination, the patient appeared ill but not toxic. Temperature was 39**°C**, and all other vital signs were normal. There were widespread, bright red, partially blanchable, discrete, and coalescing macules and papules over 75% of body surface area without blisters or erosions (Fig 1). There was bilateral conjunctival injection (Fig 2) and mild facial edema without oral mucosal involvement.

**Fig 1:**
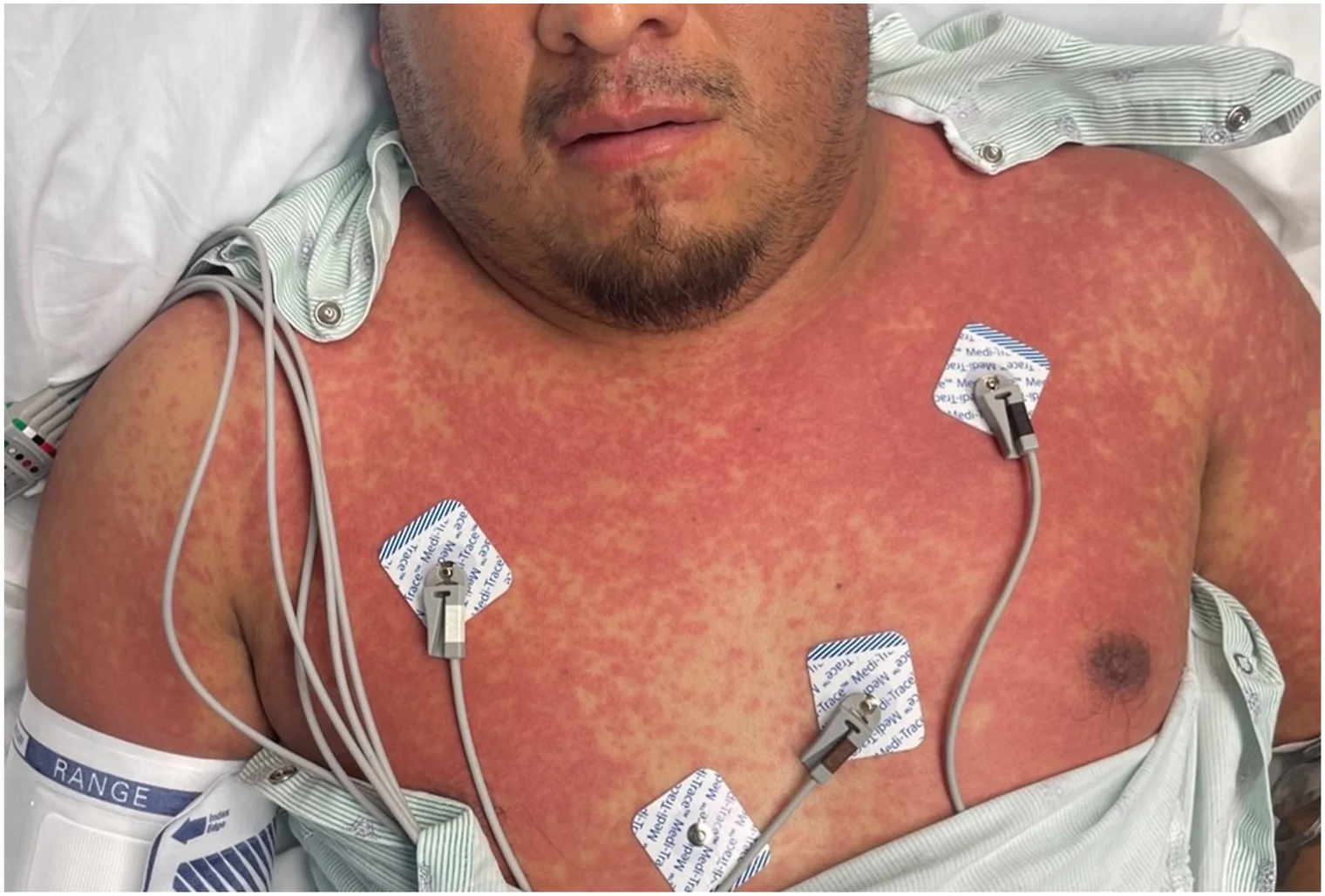
Widespread, bright red, partially blanchable, discrete, and coalescing macules and papules over 75% of body surface area without blisters or erosions present on admission.

**Fig 2:**
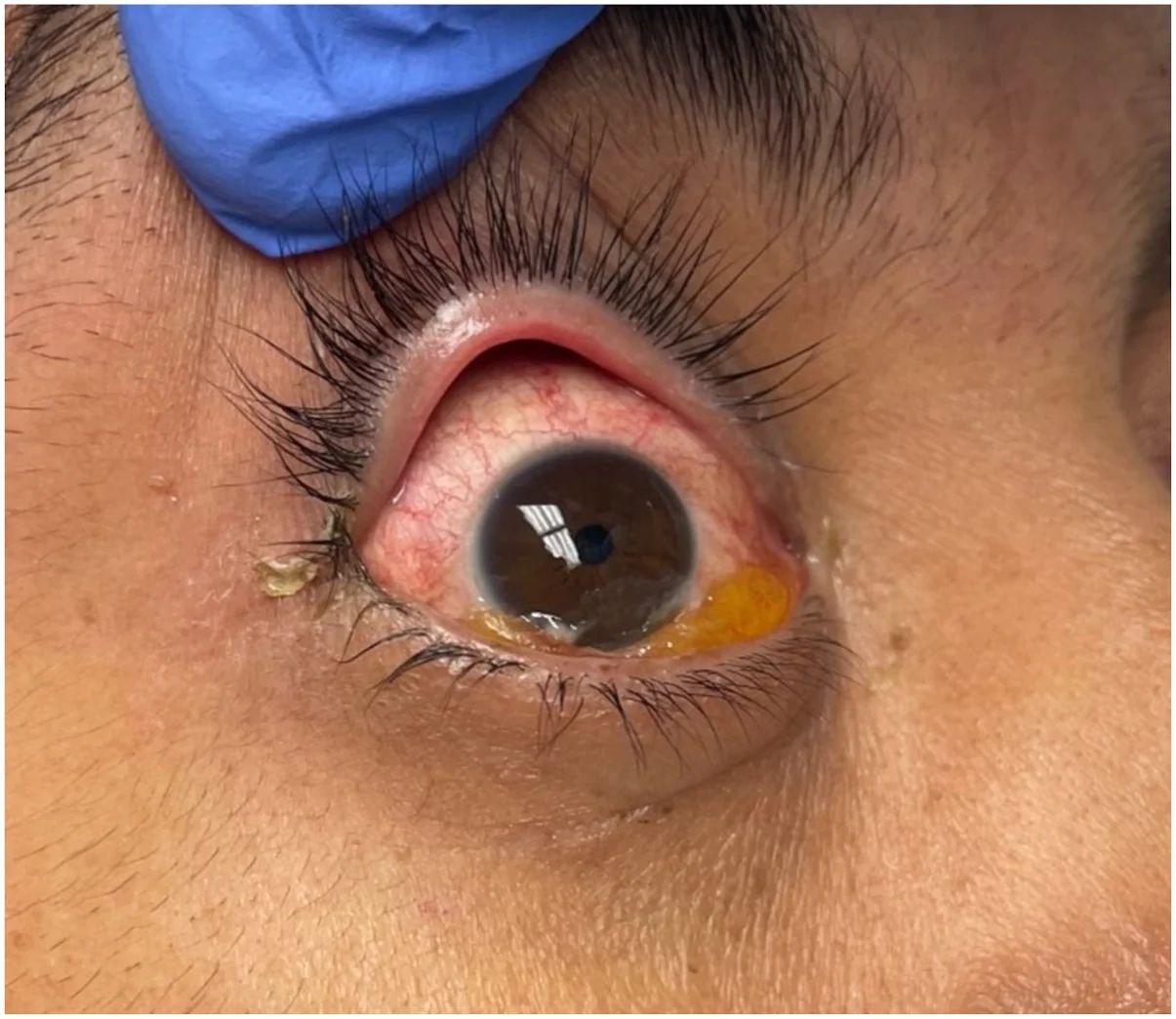
Conjunctival injection captured on hospital day two.

Laboratory studies at this time revealed normal white blood cell count (6800 cells/microliter [c/μL]), thrombocytopenia (89,000 c/μL, normal range: 150,000-450,000 c/μL), elevated liver enzymes (aspartate aminotransferase: 66 units/liter [u/L] and alanine aminotransferase: 103 u/L, normal ranges aspartate aminotransferase: 13-39 u/L and alanine aminotransferase: 8-52 u/L), D-dimer (4684 ng/mL [ng/mL], normal range: 190-499 ng/mL), and fibrinogen (>860 mg/dL [mg/dL], normal range 159-462). Infectious disease testing was negative for measles, leptospirosis, viral respiratory pathogens, legionella, human immunodeficiency virus, tick borne illnesses including Lyme disease and Rocky Mountain spotted fever, stool bacteria panel, malaria, dengue, and syphilis. Prior to empiric antibiotic therapy and again on hospital days 9 and 15, aerobic blood cultures were drawn and were negative. A punch biopsy of the rash showed patchy, scattered necrotic keratinocytes with underlying superficial perivascular, and interstitial dermal inflammation with eosinophils and neutrophils (Fig 3).

**Fig 3:**
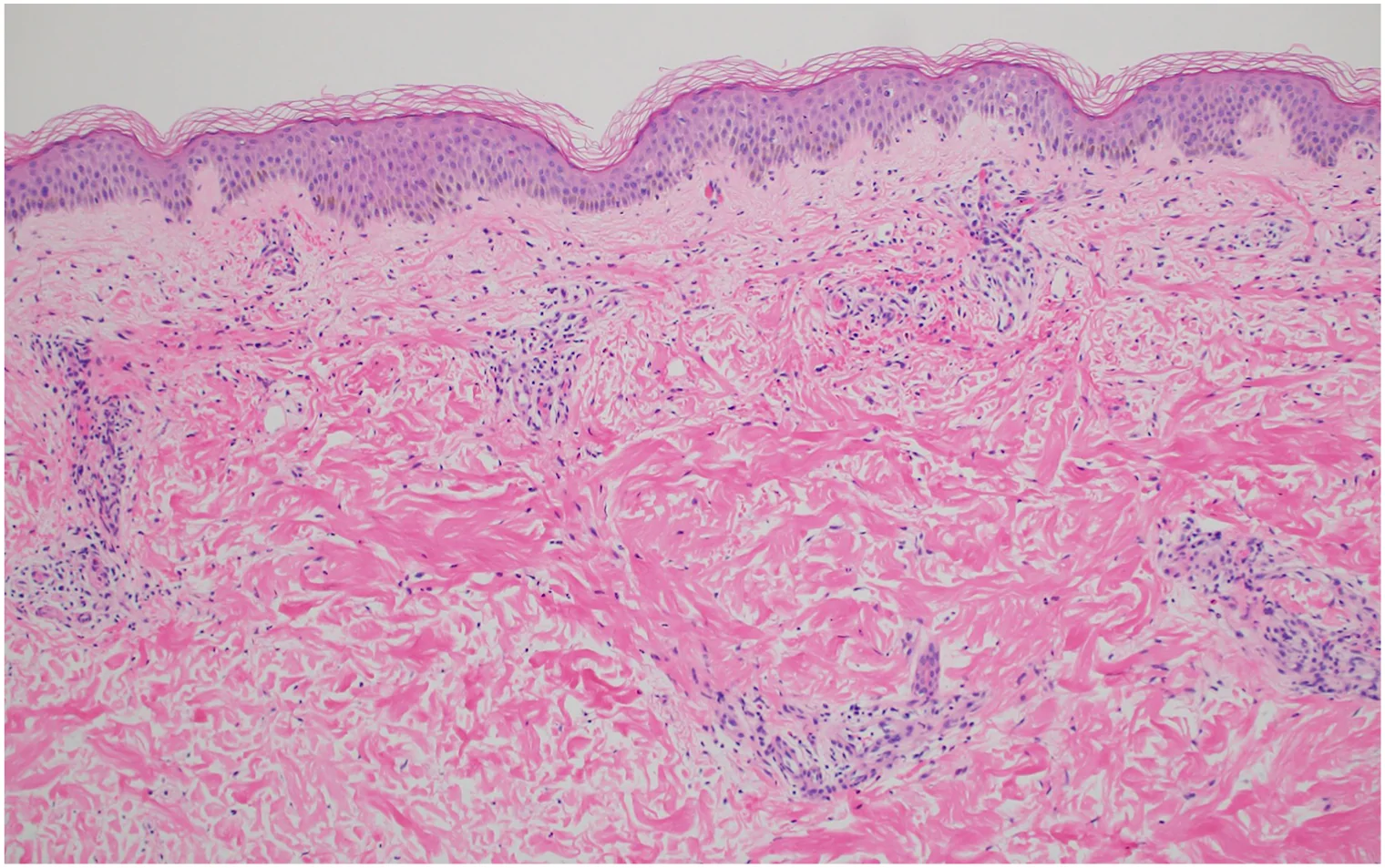
Skin punch biopsy showing patchy, scattered necrotic keratinocytes with underlying superficial perivascular and interstitial dermal inflammation with eosinophils and neutrophils (hematoxylin-eosin–stain 10×).

He was initially treated with oral doxycycline, intravenous ceftriaxone, and intravenous vancomycin. Despite this and supportive care, his status rapidly deteriorated, experiencing tachycardia, tachypnea, diffuse pulmonary edema, acute kidney injury, and oxygen desaturation requiring intubation. The patient experienced cardiac arrest on hospital day 2, febrile illness on day 7, with successful resuscitation and vasopressor support.

The rash on the lower extremities became progressively more purpuric by the end of hospital day 4 (Fig 4) associated with an increasing white blood cell count peaking at 37,000 c/μL on hospital day 5. Metronidazole was added to his antimicrobial regimen. An echocardiogram revealed a 35% ejection fraction secondary to stress/sepsis. The patient was extubated on hospital day 7 and weaned off pressor support. By hospital day 13 the rash had resolved.

**Fig 4:**
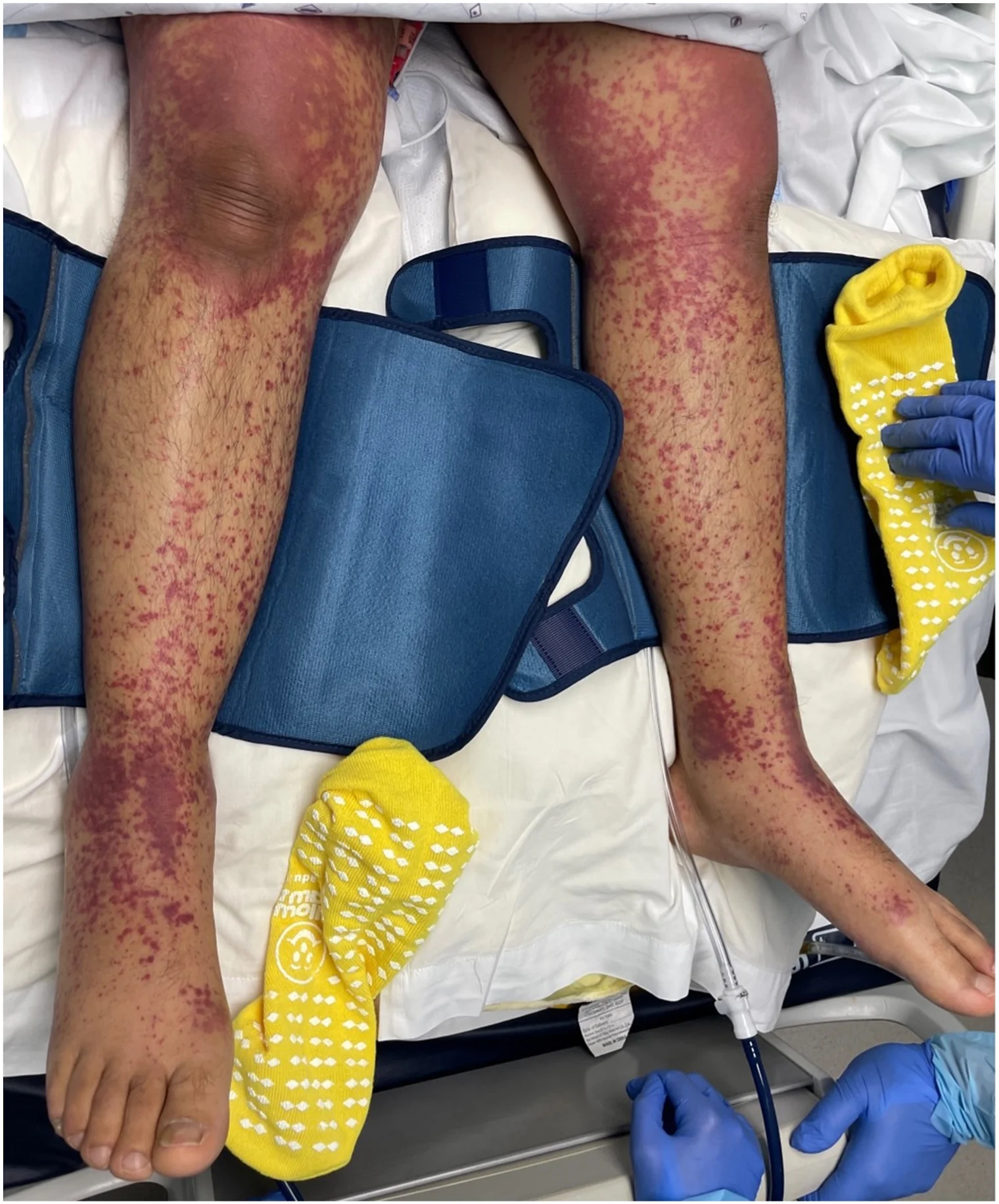
Captured on hospital day 4. Numerous deep red to purple macules, many coalescing into patches and purpura.

On hospital day 14, testing for Hantavirus IgM returned positive, prompting subtype analysis that confirmed SNV on hospital day 19. Antibiotics were discontinued and the patient recovered with residual focal unilateral facial nerve palsy. He was discharged with family support and close outpatient follow up for heart failure.

## Discussion

This patient’s course follows the classic progression of HPS: a viral prodrome with headaches, fevers, chills, and myalgias associated with thrombocytopenia and transaminitis followed by rapid deterioration of cardiopulmonary function.^2^, ^3^, ^4^, ^5^ Our patient’s infection was most likely related to his known multiple mice exposures during home remodeling. Exposure to the secretions or excretions of infected deer mice is the source of Hantavirus infection for humans.^2^^,^^4^

Wisconsin is an unusual location to diagnose SNV infection, especially in someone who has not recently traveled to a more endemic area. From 1993 to 2022, there have been 864 Hantavirus cases reported in the United States,^6^ with only 5 reported in Wisconsin. Perhaps environmental factors such as global warming are influencing northern migration of SNV vector mice, similar to northward migration patterns of the 9-banded armadillo, a vector for lepromatous leprosy.^7^ Epidemiologists and biologists should explore whether SNV is infiltrating the deer mice population in more northern states like Wisconsin.

HPS is characterized by infection of microvascular endothelial cells without significant endothelial cell destruction. Endothelial activation and the associated immune response disrupt vascular barrier integrity, resulting in increased capillary permeability. Within the lung, this microvascular leak allows protein-rich fluid to enter the pulmonary interstitium and alveolar spaces, producing noncardiogenic pulmonary edema and potentially respiratory failure.^8^^,^^9^

Cutaneous manifestations of HPS are rarely reported. In a review of 17 patients with HPS, none were reported to have conjunctival hemorrhage or purpuric rash.^1^^,^^5^ Very few reports describe definitive dermatological manifestations of SNV with clinicopathologic correlation as described in this case.

The initial erythematous eruption in our patient may represent a nonspecific viral exanthem, whereas its subsequent evolution to purpura may reflect similar microvascular dysfunction within the skin. Although the eruption became clinically vasculitic in appearance, histopathology demonstrated superficial perivascular and interstitial inflammation without evidence of small-vessel vasculitis. Thus, a purpuric or vasculitis-like eruption in HPS should not necessarily be interpreted as a true or histologically confirmable vasculitis.

Awareness of cutaneous manifestations associated with Hantavirus can aid in earlier recognition, confirmatory testing, and diagnosis. It is important to keep SNV on the differential diagnosis as a potentially emerging dangerous infectious disease.

### Declaration of generative AI and AI-assisted technologies in the writing process

None.

## Conflicts of interest

None disclosed.
